# Supplementary material for: T Cell Receptor–Major Histocompatibility Complex Interaction Strength Defines Trafficking and CD103+ Memory Status of CD8 T Cells in the Brain
Source: Front Immunol. 2018 Jun 5;9:1290. doi: 10.3389/fimmu.2018.01290 (PMC5996069; doi:10.3389/fimmu.2018.01290)

Supplementary Information

Supplementary Figure Legends

Supplementary Figure 1. pER and Ca flux upon CD3/CD28 stimulation.
A) Calcium flux assay. Lymphocytes from R7-I, R7-II or R7-III mice were preloaded with Indo-1 dye and coated with anti-CD3ε antibody. The CD3ε antibody was cross-linked with goat anti–hamster antibody at the time indicated by the break in the calcium trace. The graph shows the geometrical mean of ratio of Indo-1 violet/blue fluorescence, a measure of intracellular [Ca^{2+}], as a function of time in CD8+ T cells (representative of two independent experiments). B) pERK phosphorylation assay. Lymphocytes from R7-I, R7-II or R7-III mice were stained with α-CD4 and α-CD8 antibodies and stimulated by cross-linking of the CD3. Line graph shows the percentage of CD8 T cells that were positive for pERK at 1min intervals after cross-linking of CD3. Lines indicate the responses of CD8 T cells from R7-I (blue line), R7-II (red line) and R7-III (green line) mice. Error bars indicate SD between lymphocytes from 3 or 2 different mice.

Supplementary Figure 2. Quantification of donor R7 CD8 T cell populations at different time points post-infection.
A) Percentages of donor R7 CD8 T cells in brain, spleen, mLN and non-draining LN at 2 weeks p.i. (representative of at least 5 experiments). B) Donor R7-III CD8 T cells 5 weeks p.i. in mLN and non-draining LN are present in lower percentages (left) and absolute numbers (right) than R7-I CD8 T cells (representative of at least 5 experiments).

Supplementary Figure 3. MA plot to illustrate global transcriptional changes of peptide-activated R7 CD8 T cells.
MA plot for peptide-activated R7 CD8 T cell RNASeq samples compred to each other representing 12450 genes that had a minimum TPM value of 1 across all samples in the experiment. Genes that passed an FDR threshold <= 0.01 are highlighted in red.

Supplementary Materials and Methods

For **calcium flux** measurements lymphocytes from lymph nodes of Rop7-I, II or III mice were isolated and loaded with Indo-1 dye (Life Technologies) at concentration of 2mg/ml in IMDM media containing 5% FCS for 40 minutes at 37°C. Subsequently cells were washed 2 times with IMDM media and stained with antiCD8-Pacific Orange (5H10, Invitrogen), antiCD4-FITC (GK1.5, Biolegend) and purified antiCD3e (145-2C11, eBioscience) antibodies for 20 min at room temperature. Lymphocytes were then stimulated by addition of cross-linking antibodies against CD3 (goat anti–hamster IgG (80 mg/ml)).

For **pERK phosphorylation assay** lymphocytes from lymph nodes of R7-I, II or III mice were isolated and stained with antiCD8-Pacific Orange (5H10, Invitrogen) and antiCD4-APC (GK1.5, Biolegend) antibodies. AntiCD3e-biotin (145-2C11, eBioscience) antibody was premixed with Streptavidin RPE (Life Technologies) for 5 min at room temperature. Lymphocytes were then stimulated by addition of CD3 cross-linking mix and incubated for 0, 1, 2, 4, 8, 12 min at 37°C. At the indicated time point cells were fixed with paraformaldehyde at the final concentration of 2%. Cells were permeabilized by addition of ice-cold 90% methanol and stored over night at -20°C. Next, cells were washed and stained with anti pERK-A488 and acquired using LSR II flow cytometer. Data were analysed using Flow Jo and Prism software.

Supplementary Figure 1

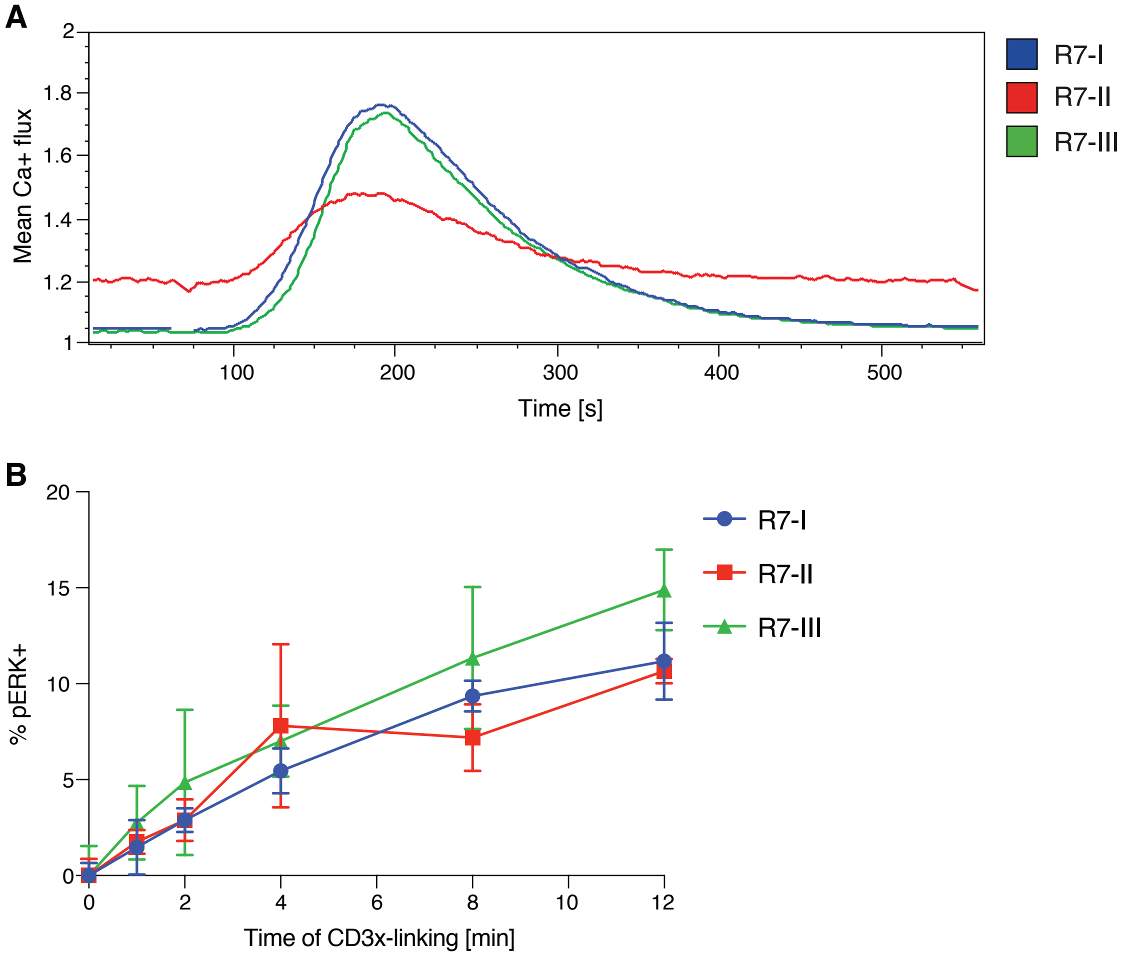

Supplementary Figure 2

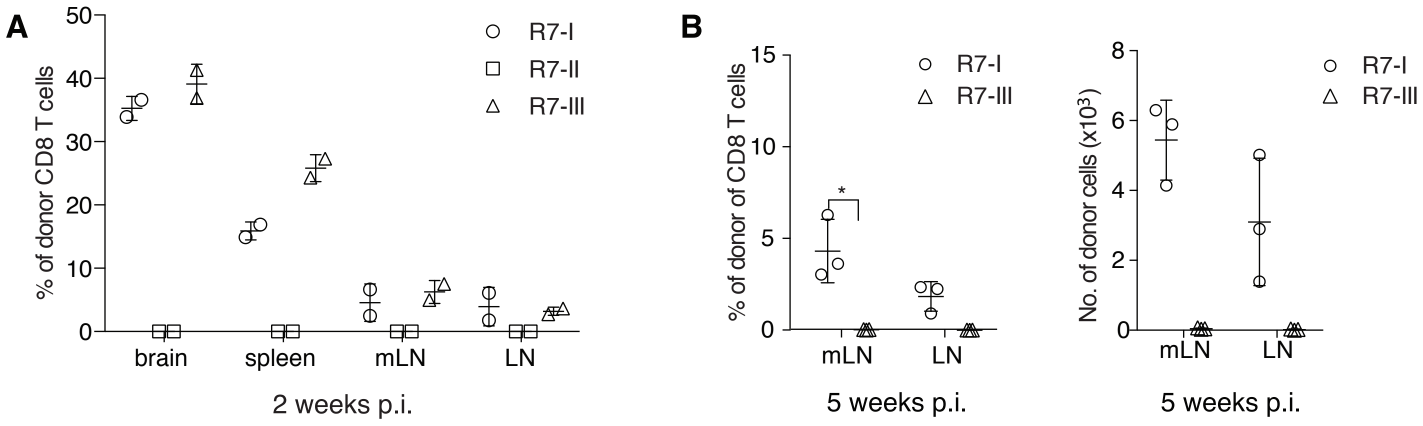

Supplementary Figure 3

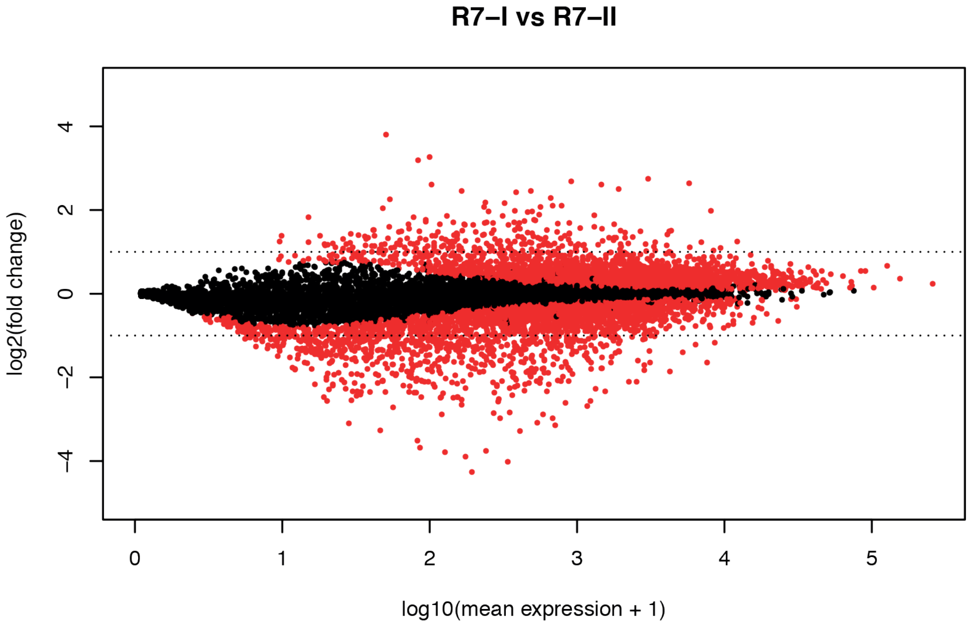

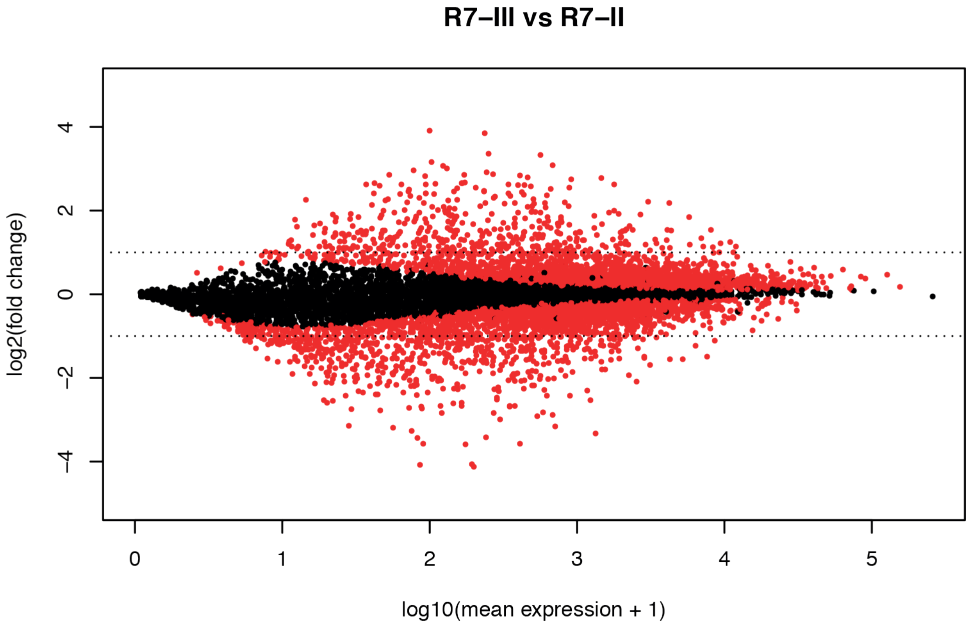

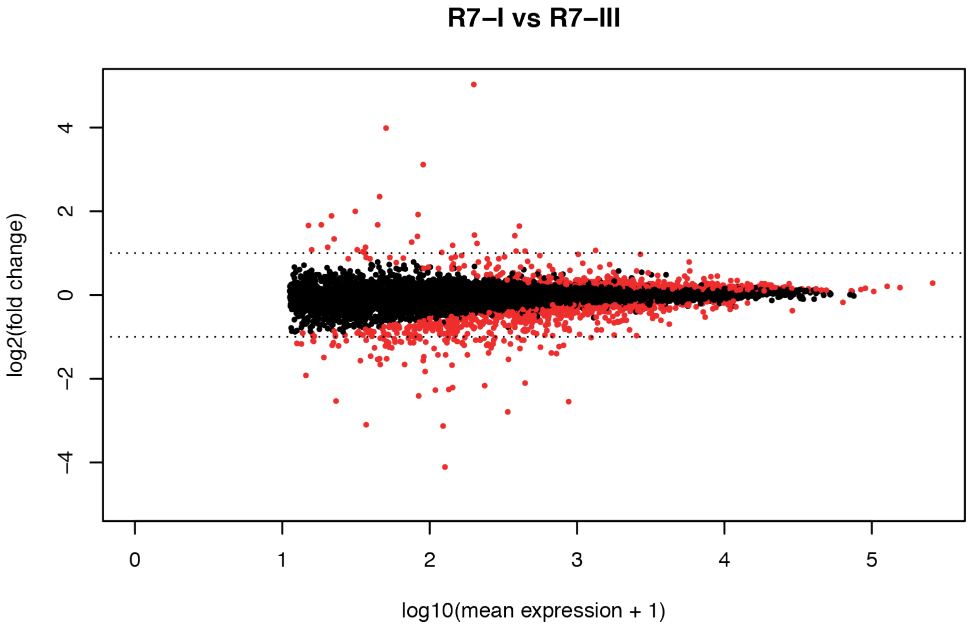

Supplement: Supplementary file 1 [file Image_1.PDF]
